# Falcon: A False Ceiling Inspection Robot

**DOI:** 10.3390/s21165281

**Published:** 2021-08-05

**Authors:** M. A. Viraj J. Muthugala, Koppaka Ganesh Sai Apuroop, Saurav Ghante Anantha Padmanabha, S. M. Bhagya P. Samarakoon, Mohan Rajesh Elara, Raymond Yeong Wei Wen

**Affiliations:** 1 Engineering Product Development Pillar, Singapore University of Technology and Design, 8 Somapah Rd, Singapore 487372, Singapore; ganesh_koppaka@sutd.edu.sg (K.G.S.A.); saurav@sutd.edu.sg (S.G.A.P.); bhagya_samarakoon@mymail.sutd.edu.sg (S.M.B.P.S.); rajeshelara@sutd.edu.sg (M.R.E.); 2 Oceania Robotics, Pte, Ltd., #01-03, 3 Soon Lee Street, Singapore 627606, Singapore; raymond@oceaniarobotics.com

**Keywords:** false ceiling inspection, inspection robotics, perimeter following, building maintenance

## Abstract

Frequent inspections are essential for false ceilings to maintain the service infrastructures, such as mechanical, electrical, and plumbing, and the structure of false ceilings. Human-labor-based conventional inspection procedures for false ceilings suffer many shortcomings, including safety concerns. Thus, robot-aided solutions are demanded for false ceiling inspections similar to other building maintenance services. However, less work has been conducted on developing robot-aided solutions for false ceiling inspections. This paper proposes a novel design for a robot intended for false ceiling inspections named Falcon. The compact size and the tracked wheel design of the robot allow it to traverse obstacles such as runners and lighting fixtures. The robot’s ability to autonomously follow the perimeter of a false ceiling can improve the productivity of the inspection process since the heading of the robot often changes due to the nature of the terrain, and continuous heading correction is an overhead for a teleoperator. Therefore, a Perimeter-Following Controller (PFC) based on fuzzy logic was integrated into the robot. Experimental results obtained by deploying a prototype of the robot design to a false ceiling testbed confirmed the effectiveness of the proposed PFC in perimeter following and the robot’s features, such as the ability to traverse on runners and fixtures in a false ceiling.

## 1. Introduction

With the growth of the population and the scarcity of land, there has been a surge in residential and corporate buildings over the past 50 years [1]. Many of these buildings have false ceilings built into their infrastructure. A false ceiling is a finished surface constructed by suspension from the actual ceiling surface with a gap of anywhere from a few centimeters to several meters [2]. They are also commonly known as dropped, suspended, and T-bar ceilings. Although it is a common contemporary fixture, false ceilings had been in use in Japan from as early as the 1300s for ceiling aesthetics [3]. Subsequently, a false ceiling was also used in Blackfriars Theater in 1596 for acoustic benefits [4]. Currently, false ceilings provide a separate space for accommodating and routing pipes, Heating, Ventilation and Air Conditioning (HVAC), and electrical wiring while allowing modular cosmetic tiling for installation [2,5,6]. In addition to that, false ceiling panels also offer acoustic treatment, moisture resistance, and fire safety characteristics [7]. Furthermore, they are simple to install, inexpensive, and serve an aesthetic purpose to the room design by hiding all of the unpleasant infrastructures to provide a homogeneous surface for the ceiling [5].

However, false ceilings come with their disadvantages, such as a weaker and less durable ceiling that reduces the height of the room ceiling [2,6]. The false ceiling can become unsanitary with humidity from the weather or moisture leaks in ducts and pipes, causing mold. Possible pest infestation is a major disadvantage since it would be difficult to eliminate these pests, most commonly rodents. Rodents flourish in false ceilings due to the lack of frequent accessibility by humans and the abundance of undisturbed space, providing them the ideal hospitable environment for breeding. Pests in false ceilings are intolerable, especially in food establishments, as they can carry the risk of spreading pathogens [8]. Pests can contaminate water and food sources, which may later contribute to spreading infectious diseases to humans. Moreover, performing maintenance or identifying issues with false ceilings is difficult since the inspection process is risky and laborious in such a confined and weak environment [9].

Robotics solutions can be seen in many building maintenance activities ranging from residential places to public and industrial places. Primarily, these robots are used in application domains such as floor cleaning [10], facade cleaning [11], gas pipes [12], and wall cleaning [13] to resolve the shortcoming of human-labor-based processes. Similarly, robotics solutions can be explored to complement the inspection process of false ceilings where the conventional human-labor-based methods suffer many shortcomings. Robots’ compact, lightweight, and agile aspects are better suited for false ceiling inspection since the works space is confined and unable to bear weight. The development of robots for inspecting confined spaces can be seen in the literature [14]. For example, the work [15] proposed a pipe inspection robot that detects defects, internal corrosion, cracks, and leakage in pipelines. In the field of cleaning, a mobile peristaltic-based duct-cleaning robot for flexible pipes was proposed that could bend as a snake while moving by contraction and expansion in the axial direction with a cleaning rate of up to 98.7% [16].

In the case of robots for ceiling inspection, there has been modest development. The Blimp robot was developed to inspect the high ceiling of a gymnasium for damage without on-site engineers [17]. Since it is a blimp robot, it is lightweight as a balloon and can access great heights efficiently with minimal energy loss. It is equipped with a WiFi camera to inspect and photograph for documentation. The work [18], proposed a compact and lightweight climbing robot that uses the hysteresis of an elastomer adhesive pad to grip a vertical wall and move around in narrow spaces. The passive peeling adhesive pad allows energy-efficient maneuvering on smooth vertical, concave, and convex surfaces. Similarly, Geckobot is a climbing robot with four peeling elastomer adhesive pads that can climb irregular terrain [19]. This robot even has an active tail to distribute weight and balance better. Another quadruped robot called UNIclimb [20] was developed using bio-inspired dry adhesive pads, SLS-type 3D printing, and super-hydrophobic coating that proved to be effective even for inverted maneuvers on a horizontal ceiling. The coating enabled it to work in wet, humid, and raining environments as well. Besides bio-inspired adhesive pads, a magnet-based adhesive wall and ceiling climbing robot was developed to maneuver ferromagnetic surfaces such as steel cargo containers for contraband inspection using permanent magnetic wheels [21]. In terms of the inspection and damage assessment methodology, a smart sensor board was developed for ceiling damage detection [22]. This sensor board can be integrated into a wheel-type or crawler-type inspection robot and connected to a wireless network for remote operations. A false ceiling inspection robot must traverse inside components such as runners and wires during the inspection process. Furthermore, the robot should be capable of accessing confined spaces and be lightweight. However, the design of a robot specifically targeted for false ceilings inspections has not been explored yet.

This paper proposes a novel robot design intended for false ceiling inspections. The proposed robot has a compact, lightweight design that allows it to move through confined spaces. The locomotion system was designed to enable the robot’s ability to traverse components found in typical false ceilings such as runners and cables. Furthermore, the robot is integrated with the autonomous perimeter-following functionality to improve the productivity of the inspection process. A comparison between the main capabilities of the proposed robot, Falcon, and other existing robots is given in Table 1 to highlight the advantages of the proposed design. The design requirements of a false ceiling inspection robot are scrutinized in Section 2 considering typical inspection processes. Section 3 details the design of the robot. The proposed Perimeter-Following Controller (PFC) is explained in Section 4. Section 5 discusses the experimental validation of the proposed robot design and the PFC. Conclusions of the work are given in Section 6.

## 2. Design Requirement for a False Ceiling Inspection Robot

**Figure 1:**
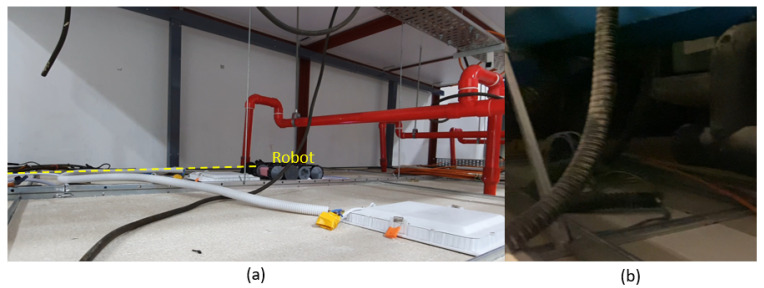
Robot-aided inspection process of a false ceiling. (**a**) A robot inspecting a false ceiling. The dashed line represents a path preferred by the robot for inspection. (**b**) Poor lighting condition expected in a false ceiling.

A scenario of a robot inside a false ceiling is depicted in Figure 1. False ceilings are used to conceal the service lines of buildings such as mechanical, electrical, and plumbing services to improve the appearance. These include components related to firefighting systems, such as sprinkler water supply and smoke detectors, components of electrical wiring, such as cables and lighting fixtures, and ducts of ventilation systems. Frequent inspections are essential to ensure the proper functioning of these service infrastructures. In addition to that, the maintenance issues might cause progressive damage to the ceiling structures. For example, water leakages can cause progressive damage to the structure. Pest and rodent infestations also damage the equipment of service infrastructures and the ceiling structure. However, these maintenance requirements are harder to notice since the ceiling panels conceal all the components; inspection should be conducted by deploying a robot in a false ceiling. In this regard, the following features are essentially required for a false ceiling inspection robot:

- The space availability is limited inside a false ceiling, and a robot often has to navigate narrow and low-height passages. Therefore, the robot should have a compact size that enables it to move through narrow passages;
- The surface of a false ceiling consists of runners, lighting fixtures, electrical cables, and ducts. Therefore, the robot should have the ability to traverse these components to cover a false ceiling during the inspections;
- False ceilings are hanged from the main structures by cables. In addition to that, the ceiling panels are made from low-strength materials where the weight bearability has limitations. Therefore, the robot should be lightweight;
- In the inspection process, it is necessary to move the robot along the perimeter to observe the inside of a false ceiling correctly. However, it is not easy to move the robot along a perimeter merely using teleoperation. The continuous heading variation of the robot due to terrain conditions is the main reason for this difficulty. Thus, the ability to autonomously follow the perimeter is crucial to improve the robot-aided inspection process of a false ceiling;
- The robot should be equipped with a vision system to provide visual feedback of the inside environment. This vision system should be capable of working in low-light conditions. Moreover, the robot should be equipped with an illumination source to complement the vision system.

## 3. Robot Platform

**Figure 2:**
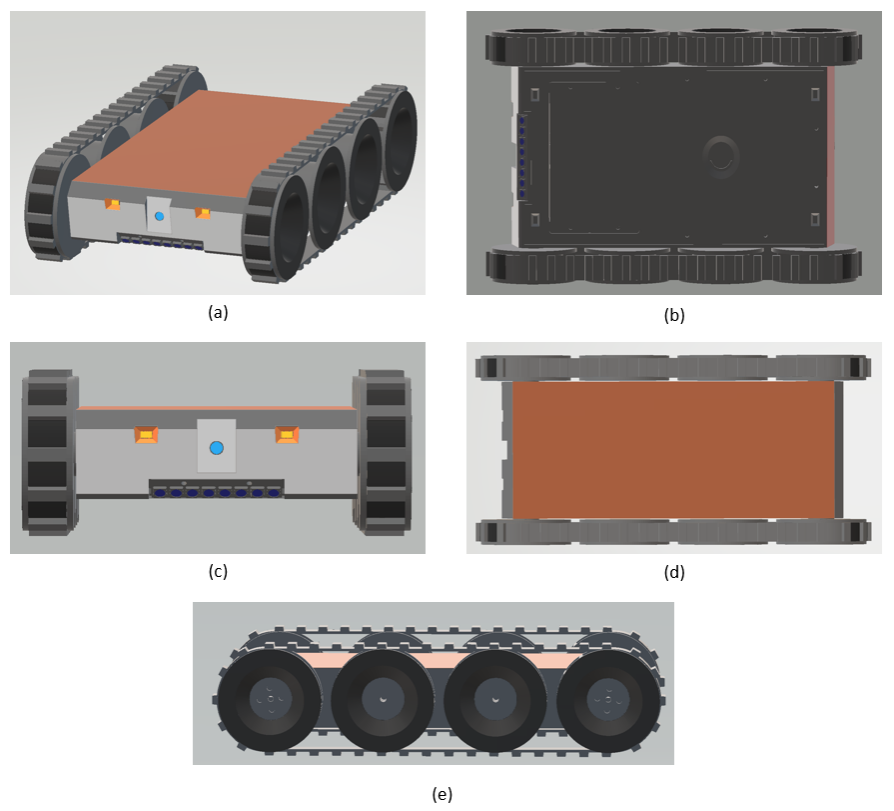
Design of the robot. (**a**) Isometric view; (**b**) bottom view; (**c**) front view; (**d**) top view; (**e**) lateral view.

**Figure 3:**
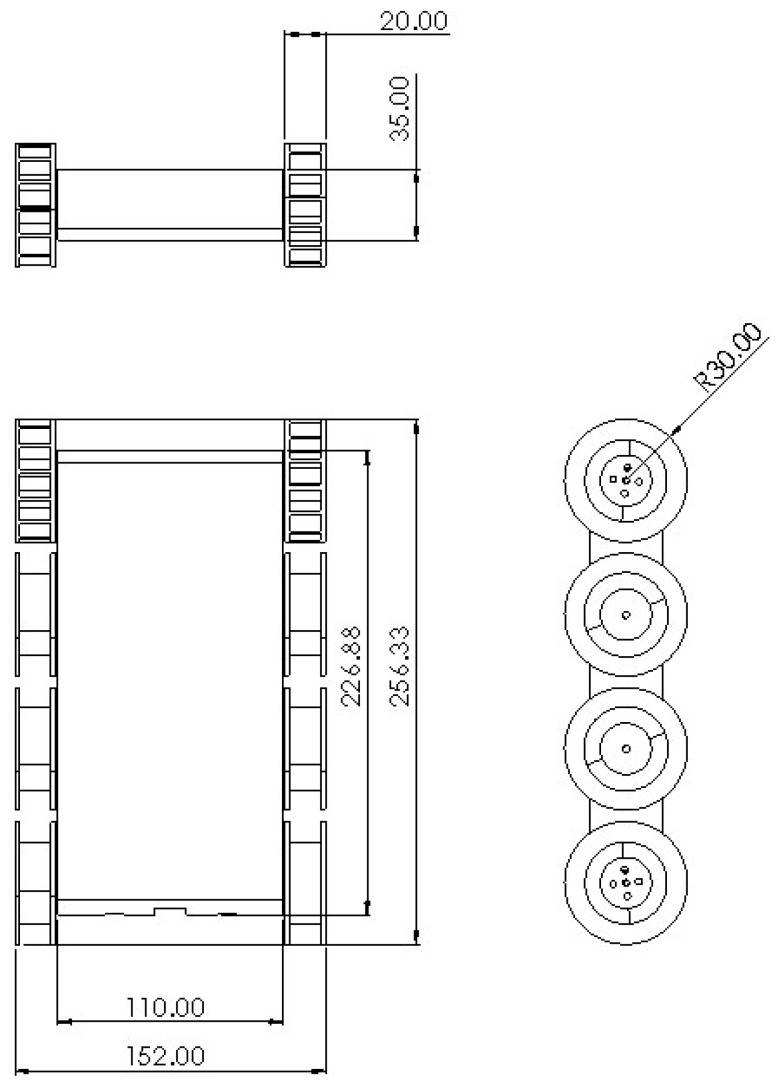
Dimensions of the robot.

The robot proposed in this paper is named “Falcon”. Falcon is a modular and scalable robot designed and developed for false ceiling inspections, among other tasks. The robot was designed on a modular basis that allows it to address complexities in the areas that might cause hindrances to locomotion and task execution. As the interior of the false ceilings is usually filled with various fixtures, such as runners and cables, it is important to consider the locomotion aspects of the robot. The locomotion design of the robot utilizes a tracked drive, which allows the robot to move in difficult terrain conditions to address the requirement mentioned above. The material for the track was chosen to be rubber as it offers better friction to have a good grip. There are four DC motors, two on each side, secured inside the robot that come in contact with the treads, thus enabling them to actuate. The 3D-rendered images of the Falcon robot are illustrated in Figure 2. The robot weighs about 1.5 kg with dimensions spanning approximately 152 mm (L) × 257 mm (W) × 60 mm (H). The dimensions of the robot are detailed in Figure 3. A front-facing monocular camera with an LED-based illumination source is attached to the robot to facilitate the inspections.

### 3.1. Mechanical Design

The robot body and the wheels were 3D printed with Poly-Lactic Acid (PLA) material. This material offers lightweight and strong features to the robot. The robot materialized from the design after keenly considering the design arrangements inside a typical false ceiling setup. The current robot has a tracked skid steering mechanism. The locomotion of the robot is controlled by controlling the relative velocities of both tracks. Due to the similar steering mechanisms of the wheeled and the tracked skid steering, a wide range of their properties overlap.

**Figure 4:**
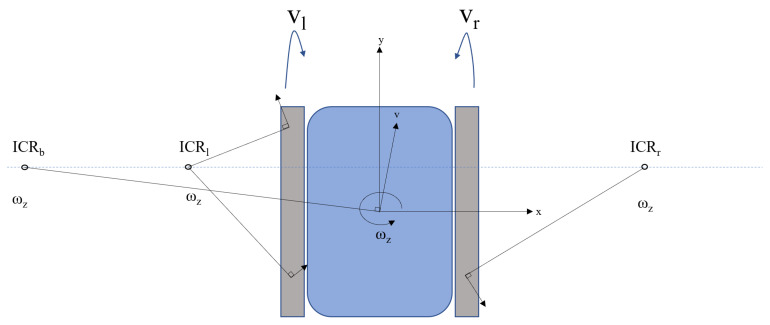
The locations of the ICRs as the robot makes a circular turn with angular and linear velocities.

For the ease of understanding, a simple skid steering kinematics was used in this paper. There were certain assumptions made for the kinematic presentation, such as the center of the mass being at the center of the robot, no difference in the speeds of the motor drives, and the robot runs on a flat surface with all the drive wheels in contact with the surface. The kinematic model of the robot was derived based on Figure 4.

Consider two frames of reference: one is the global frame {X,Y} and the other is the robot frame $\left\{x^{R},y^{R}\right\}$. As the goal was to find the relative velocities of the wheel drives of the robots, the relation between the velocities in the global frame and the robot frame is given in (1) by considering homogeneous transformation between the frames [23]. The linear velocity in the local frame is given as ${\left[v_{x},v_{y},0\right]}^{T}$ and the angular velocity as ${\left[0,0,\omega_{z}\right]}^{T}$. Here, ${\left[p,q,\dot{\theta }\right]}^{T}$ is a vector representing generalized velocities.

(1) $$\left[\begin{array}{c} p\\ q\\ \dot{\theta } \end{array}\right]=\left[\begin{array}{ccc} cos\theta & -sin\theta & 0\\ sin\theta & cos\theta & 0\\ 0 & 0 & 1 \end{array}\right]\left[\begin{array}{c} v_{x}\\ v_{y}\\ \omega_{z} \end{array}\right]$$

As the robot moves, its tread velocities are calculated based on the Instantaneous Centers of Rotation (ICRs). Here, there are three ICRs, namely $ICR_{l}$, $ICR_{r}$, and $ICR_{b}$, which are the ICR of the left tread, the ICR of the right tread, and the ICR of the robot body, respectively. Their coordinates are denoted as $\left(x_{l},y_{l}\right)$, $\left(x_{r},y_{r}\right)$, $\left(x_{b},y_{b}\right)$, respectively. Considering that the treads and the robot body both have the same angular velocity $\omega_{z}$, the relation among the ICR and the velocities can be given by (2)–(5). Here, *r* is the radius of the wheel to which the tread is connected and $v_{r}=\omega_{r}r$ and $v_{l}=\omega_{l}r$.

(2) $$x_{l}=\left(\omega_{l}r-v_{y}\right)/\omega_{z}$$

(3) $$x_{r}=\left(\omega_{r}r-v_{y}\right)/\omega_{z}$$

(4) $$x_{b}=-v_{y}/\omega_{z}$$

(5) $$y_{b}=y_{l}=y_{r}=v_{x}/\omega_{z}$$

Equations (2)–(5) help estimate the inverse functions. With respect to the robot frame of reference, the translation speed and the rotational speed are given by (6)–(8).

(6) $$v_{x}=\frac{v_{r}-v_{l}}{x_{r}-x_{l}}\times y_{l}$$

(7) $$v_{y}=\frac{v_{r}+v_{l}}{2}\;-\frac{v_{r}-v_{l}}{x_{r}-x_{l}}\left(\frac{x_{l}+x_{r}}{2}\right)$$

(8) $$\omega_{z}=\frac{v_{r}-v_{l}}{x_{r}-x_{l}}$$

These equations hold well for the robot kinematics when the ICRs of the left and right treads are estimated correctly. The inverse kinematics relations can be used to find the individual tread velocities to be issued for moving the robot per the given velocity command. These relations are given in (9) and (10).

(9) $$v_{r}=v_{y}+x_{r}\times \omega_{z}$$

(10) $$v_{l}=v_{y}+x_{l}\times \omega_{z}$$

As with the presence of the track slip, the ICRs of the tracks always lie outside of the robot. If there is an ideal slip situation, then the ICRs of the tracks lie closer to the robot. This behavior is also dependent on the Center Of Mass (COM) concentration of the robot. Based on the position of the COM, the positions of the ICRs will be either close to the robot or far away because of the pressure distribution, which affects the area of contact.

### 3.2. Electrical Design

**Figure 5:**
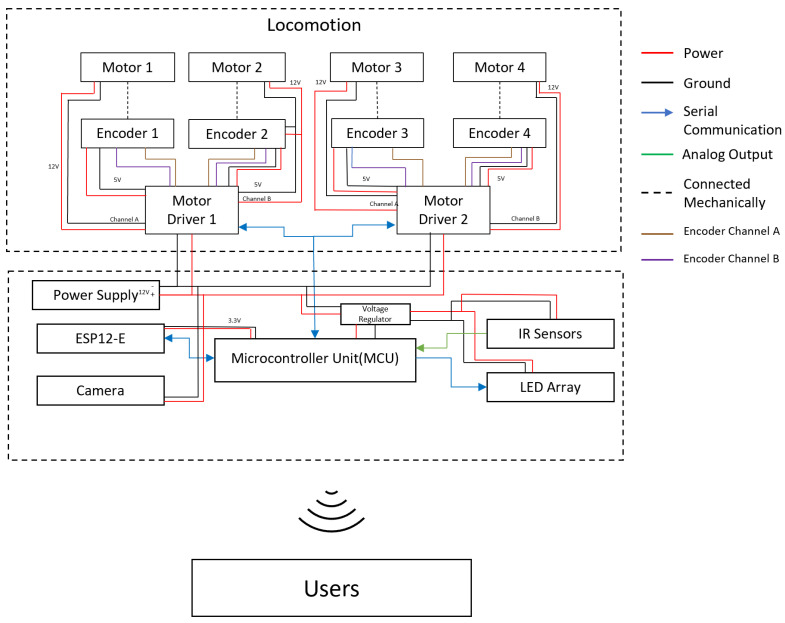
Overview of the electronic system.

The electronic subsystem of the robot is depicted in Figure 5. The robot has a Microcontroller Unit (MCU) (Arduino Mega2560, https://store.arduino.cc/usa/mega-2560-r3 accessed on 25 June 2021), which acts as the brain of the robot. It houses all the logic required for the robot to perform the inspection tasks. It also has an ESP12-E module (WEMOS D1 Mini, https://www.wemos.cc/en/latest/d1/d1_mini.html accessed on 25 June 2021) that functions as a slave, enabling the WiFi capability of the robot. The DC motors, https://www.pololu.com/product/3046 accessed on 25 June 2021 (35 rpm nominal speed, 60 mA no-load current, 986.41:1 gear ratio, 10 kgcm stall torque at 12 V) are driven through two motor drivers (RoboClaw 2x7A dual-channel motor driver, https://www.basicmicro.com/Roboclaw-2x7A-Motor-Controller_p_55.html accessed on 25 June 2021). The MCU issues the respective motor velocity commands for the robot through motor drivers according to the necessary control actions. For precise locomotion, it is vital to have a feedback mechanism. Therefore, the DC motors are also equipped with encoders (magnetic encoder with 12 counts per revolution, https://www.pololu.com/product/4761 accessed on 25 June 2021) that help in coupling the feedback mechanism to the locomotion. The robot also houses an array of Infrared (IR) sensors (Sharp GP2Y0A41SK0F, https://global.sharp/products/device/lineup/data/pdf/datasheet/gp2y0a41sk_e.pdf accessed on 25 June 2021) to detect the closest obstacle and functionalities such as perimeter following. The IR sensors are connected to the MCU through analog-to-digital converter pins. The entire electronic setup was fabricated on a printed circuit board for easy mounting. Furthermore, it was designed with the provision for the integration of auxiliary components such as sensor and actuator modules. The robot is powered up by a lithium ion battery pack (12 V, 3000 mAh). A step-down voltage regulator (Pololu 5 V, 5 A step-down voltage regulator D24V50F5, https://www.pololu.com/product/2851 accessed on 25 June 2021) was also used to power up the components working on 5 V such as the MCU and IR sensors.

The video (Supplementary Material) feed of the monocular camera (Trek Ai-Ball) is streamed through WiFi to a remote terminal where an operator can perform real-time inspections. In addition, as the lighting conditions in the false ceiling are insufficient, an LED strip (NeoPixel Stick, 8 × 5050 RGB LED) is also attached to the robot, which acts as an illumination source. The color component (RGB) and brightness of each LED of the strip can be individually controlled through the Arduino. The brightness controllability would help the inspection. Furthermore, different color schemes can be used to indicate the different statuses of the robot. With the current configuration of the camera mechanism, an operator can view an area within a distance range of about 60 cm to 70 cm with a field of view of about 60${}^{\circ }$ in both axes. The video stream of this camera was expected to be primarily utilized for inspection. Furthermore, the robot was designed to install additional sensors and actuators such as olfactory sensors and rodenticide dispensers for complementing the inspection. In this regard, the platform was designed with the provision to operate with an additional weight of 200 g stably. At this stage, the robot has to be teleoperated for inspection, and no autonomous functionality other than the autonomous perimeter-following ability was implemented on the robot.

## 4. Perimeter-Following Controller

**Figure 6:**
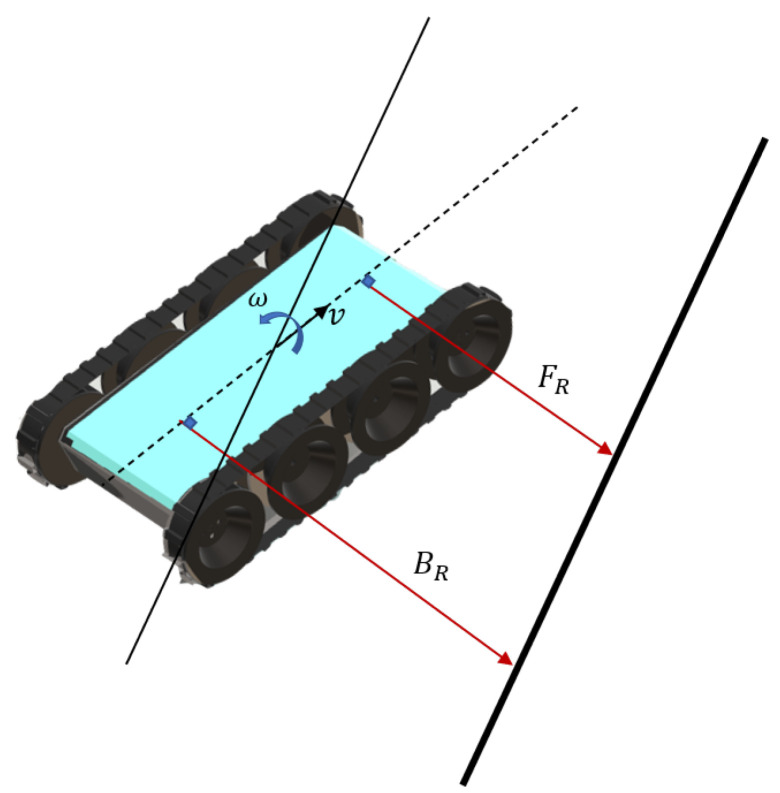
Robot sensing a perimeter wall. The front range and bottom range are given as $F_{R}$ and $B_{R}$, respectively. The angular velocity and linear velocities are given as *w* and *v*, respectively.

A scenario where the Falcon robot follows a perimeter wall is represented in Figure 6. The robot perceives the distances from the perimeter with the IR sensors in the front and back as $F_{R}$ and $B_{R}$, respectively. A difference between $F_{R}$ and $B_{R}$ indicates that the robot heading is not parallel to the wall. The robot should change the angular velocity (i.e., ω) to correct its heading error to be parallel with the perimeter when the robot moves with a fixed linear velocity (i.e., *v*). The error of the robot heading $e_{o}$ can be estimated as $e_{o}=B_{R}-F_{R}$. The controller should attempt to take actions to minimize this error during the task of perimeter following. The Perimeter-Following Controller (PFC) of the robot was developed using fuzzy logic. Fuzzy logic was utilized to implement the PFC due to the following reasons:

- Fuzzy logic can be used to map the input and output space of a process using a set of linguistic rules without the knowledge of the underlying dynamics of the process model [24,25,26]. In the case of the Falcon robot, the exact dynamics of the robot are not known due to the difficulty of modeling the slippage of the robot when traversing on bumps such as runners and cables;
- Range sensor information inside a confined space, such as inside a false ceiling, is not accurate [27,28]. Thus, the proposed robot often encounters sensor inaccuracies where the decisions have to be made while coping with the sensor uncertainties. Fuzzy logic is effective for decision making based on such inaccurate sensor measurements [29,30];
- The environment inside a false ceiling is uncertain due to the inclusion of cluttered objects such as cable trunks, air ducts, and runners. Fuzzy logic has been proven to be effective in navigating such uncertain environments [31,32];
- Expert knowledge can easily be modeled using fuzzy logic [33,34]. Thus, the knowledge of a robot operator could be utilized for modeling the required decision-making behavior of this scenario.

**Figure 7:**
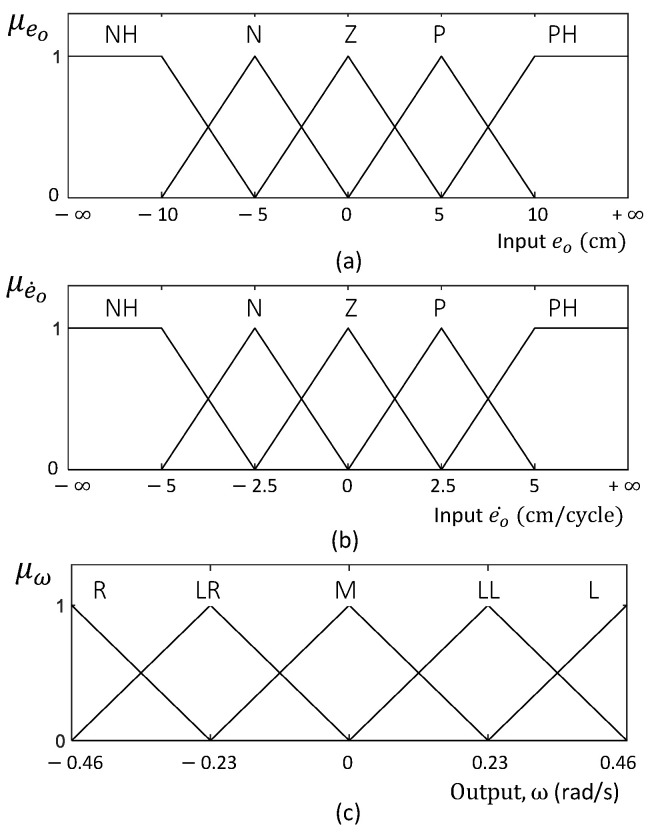
(**a**,**b**) represent the input membership functions. The range of these membership functions extends from −∞ to +∞. (**c**) represents the output membership function of the fuzzy logic system. The fuzzy labels are defined as NH: Negative High, N: Negative, Z: Zero, P: Positive, PH: Positive High, R: Right, LR: Little Right, M: Medium, LL: Little Left, and L: Left.

The inputs of the PFC are the error in the orientation of the robot ($e_{o}$) and the change of error in the orientation (${\dot{e}}_{o}$). The output of the fuzzy logic system is the angular velocity of the robot (ω). The input and output membership functions of the fuzzy logic system are shown in Figure 7. Triangular and trapezoidal fuzzy sets were defined for the membership functions to reduce the computational resources required for the inferencing [35]. Furthermore, this facilitates the simplicity and the efficiency of the developed system implementation. The fuzzy set ranges were defined such that they evenly covered the universe of discourse of the input and output spaces. The universe of discourse of the inputs and the outputs were experimentally determined considering the probable variations of the inputs and the feasible range of controlling the output. However, the universe of discourse of the inputs was extended from −∞ to +∞ to cope with any input value that might be caused due to noises. The number of fuzzy rules required for a fuzzy logic controller depends on the number of input and output membership functions. Although there is no exact limit to the number of fuzzy sets for a given membership function, Wu et al. [36] emphasized that membership functions should be limited to seven for efficiency and the proper representation of expert knowledge. Considering the above details, five fuzzy sets were used for the input and output membership functions, which covered the input and output spaces with a sensitivity sufficient for the controller.

$\mu_{e_{o}}\left(e_{o}\right)$ and $\mu_{{\dot{e}}_{o}}\left({\dot{e}}_{o}\right)$ correspond to the fuzzified inputs of the system. The linguistic rules defined based on expert knowledge are given in Table 2. These rules illustrate the decision making of control actions and the policy of the fuzzy logic system. The rules were defined in a way that mapped the input space toward the output space. Minimum and maximum operators were used as the t-norm and t-conorm of the fuzzy logic system, respectively. Hence, the firing strength of the *i*th rule, $\Gamma_{i}$, can be defined as in (11).

(11) $$\Gamma_{i}=\min\left\{\mu_{e_{o_{i}}}\left(e_{o}\right),\mu_{{\dot{e}}_{o_{i}}}\left({\dot{e}}_{o}\right)\right\}$$

The output fuzzy consequent of the *i*th rule can be obtained as in (12) by applying the Mamdani implication method. Here, $\mu_{\omega_{i}^{\prime }}$ is the fuzzy consequent resulting from clipping the output fuzzy set by the corresponding firing strength. For the fuzzy aggregation operator, the maximum was used as in (13) to combine the fuzzy consequents into a single set where *N* is the number of rules in the rule base. The crisp output to control the robot’s reference angular velocity, ω*, for effective perimeter following can be obtained as in (14) considering the center of area defuzzification method.

(12) $$\begin{array}{c} \mu_{\omega_{i}^{\prime }}\left(\omega \right)=\min\left\{\Gamma_{i},\mu_{\omega_{i}}\left(\omega \right)\right\} \end{array}$$

(13) $$\begin{array}{c} \mu_{\omega^{\prime }}\left(\omega \right)=\max\left\{\mu_{\omega_{1}^{\prime }}\left(\omega \right),\mu_{\omega_{2}^{\prime }}\left(\omega \right),\ldots ,\mu_{\omega_{i}^{\prime }}\left(\omega \right),\ldots ,\mu_{\omega_{N}^{\prime }}\left(\omega \right)\right\} \end{array}$$

(14) $$\begin{array}{c} \omega *=\frac{\int \omega \mu_{\omega^{\prime }}\left(\omega \right)d\omega }{\int \mu_{\omega^{\prime }}\left(\omega \right)d\omega } \end{array}$$

## 5. Experimental Validation

### 5.1. Experimental Setup

A prototype of the proposed robot was developed. The experiments were conducted in two phases. The robot’s ability to traverse the components inside a false ceiling, such as runners, cables, and lighting fixtures, was tested in the first phase since it is an essential design consideration for a false ceiling inspection robot. In the second phase, the performance and behavior of the proposed PFC were evaluated considering a set of heterogeneous test cases. All the experiments were conducted on a mock setup of a false ceiling constructed per the specifications of typical false ceilings. The linear velocity of the robot (i.e., *v*) was configured to 0.06 m/s for the experiment. The time step of the control loop was 64 ms.

### 5.2. Verification of the Obstacle Traversing Ability of the Robot

**Figure 8:**
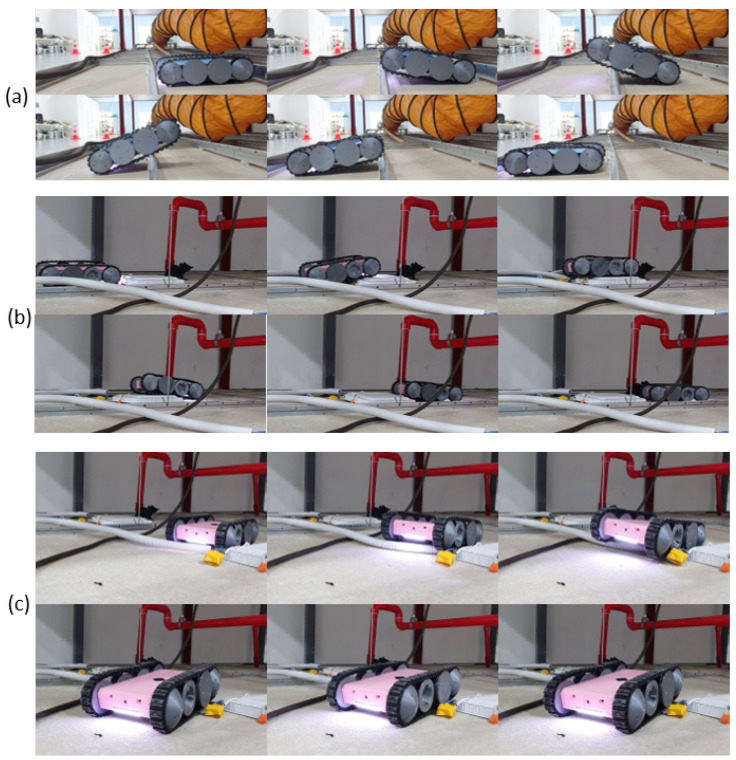
Sequence of snapshots taken when the robot traverses different components of a false ceiling. (**a**) Moving across a runner; (**b**) moving on a lighting fixture; and (**c**) crossing a conduit.

The ability to traverse components inside a false ceiling, such as runners, cables, conduits, and lighting fixtures, was one of the essential design features expected from the proposed robot design. Therefore, the robot’s ability to traverse these sorts of obstacles was evaluated considering typical false ceiling structures. In this regard, the robot was moved on the objects multiple times. Sequences of snapshots taken during sample test cases are given in Figure 8. According to the experimental observations, the robot was able to traverse through the objects as expected in the design process. These observations validated that the proposed robot design enables it to traverse common objects in a false ceiling, and thus, it would be helpful for the inspection process.

### 5.3. Performance and Behavior of the PFC

**Figure 9:**
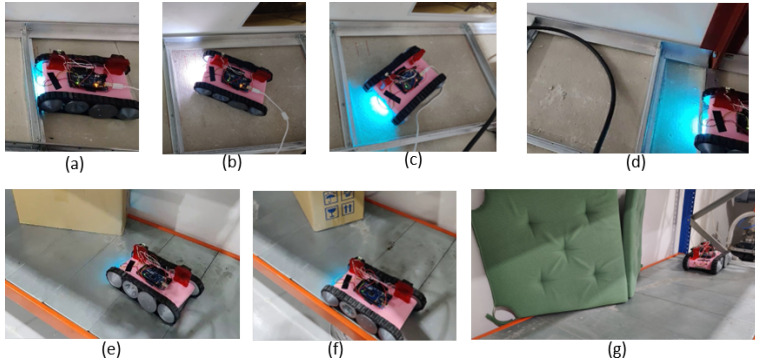
The arrangements of the test cases used in the second phase of the experiment. (**a**) The robot was placed with no heading error with the perimeter wall. (**b**) The robot was placed with an inward heading error. (**c**) The robot was placed with an outward heading error. (**d**) A segment with more objects to be traverse. (**e**) A segment with an inward slanting perimeter. (**f**) A segment with an outward slanting perimeter. (**g**) A segment with an inward and outward slanted perimeter.

**Figure 10:**
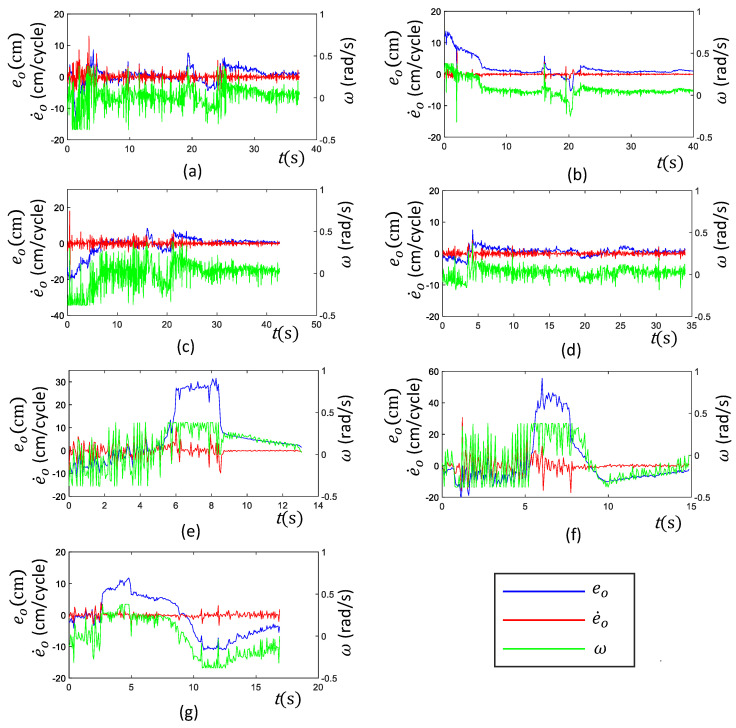
Variation of the inputs and output of the PFC corresponding to the test cases explained in Figure 9. It should be noted that the Y-axis corresponding to ω is on right side of each plot. The corresponding test cases are indicated by the subfigures as follows: (**a**) The robot was placed with no heading error with the perimeter wall. (**b**) The robot was placed with an inward heading error. (**c**) The robot was placed with an outward heading error. (**d**) A segment with more objects to be traverse. (**e**) A segment with an inward slanting perimeter. (**f**) A segment with an outward slanting perimeter. (**g**) A segment with an inward and outward slanted perimeter.

**Figure 11:**
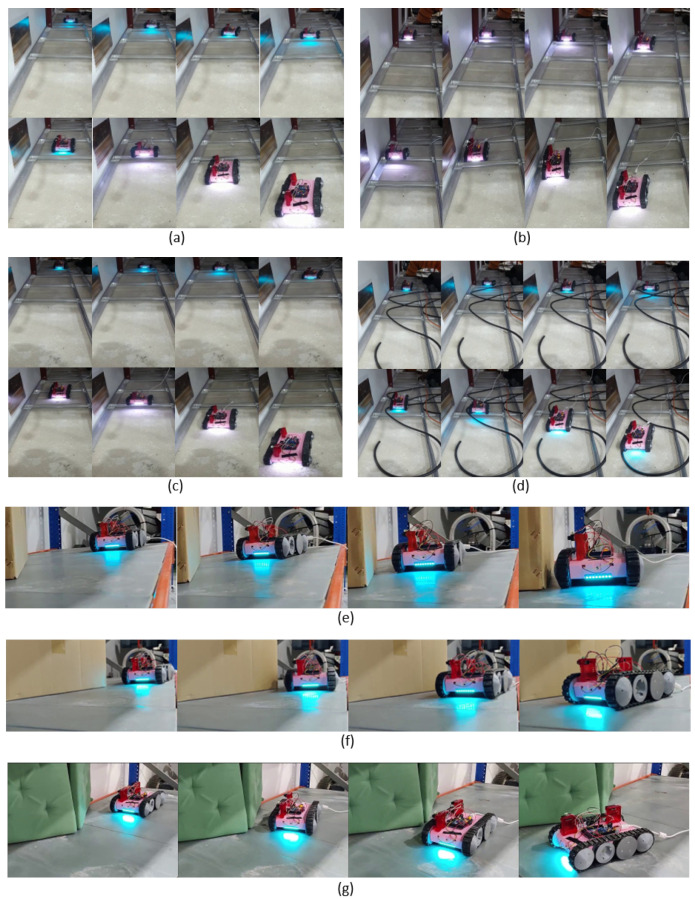
Snapshot sequences of the robot’s movements during the test cases explained in Figure 9. The snapshots displayed here for a test case are evenly distributed within the duration of the case. (**a**) The robot was placed with no heading error with the perimeter wall. (**b**) The robot was placed with an inward heading error. (**c**) The robot was placed with an outward heading error. (**d**) A segment with more objects to be traverse. (**e**) A segment with an inward slanting perimeter. (**f**) A segment with an outward slanting perimeter. (**g**) A segment with an inward and outward slanted perimeter.

Seven heterogeneous test cases were considered to verify the performance and the behavior of the proposed PFC. The arrangement of those seven test cases is shown in Figure 9. The inputs and the output of the PFC (i.e., $e_{o}$, $\dot{e_{o}}$, and ω) were logged for all the cases. The observed variations of these parameters during the test cases are given in Figure 10. Furthermore, the robot’s movement was recorded through an external camera, and a sequence of snapshots of the robot for each corresponding test case is given in Figure 11. The variation of the robot’s heading with respect to the perimeter wall (i.e., $e_{o}$) was crucial for assessing the performance. Thus, the Root Mean Square (RMS) of $e_{o}$ was used to quantitatively analyze the overall performance of the PFC in a test case. Here, the RMS of $e_{o}$ was used instead of the mean since positive and negative values of $e_{o}$ nullify the overall error. The RMS of $e_{o}$, $RMS_{e_{o}}$, was calculated as in (15), where *T* is the duration of a test case. $RMS_{e_{o}}$ observed for each case are given in Table 3.

(15) $$RMS_{e_{o}}=\frac{1}{T}\sum_{t=0}^{T}e_{o}^{2}\left(t\right)$$

In Test Case a, the robot was initially placed without a heading error with the perimeter wall as shown in Figure 9a, and the Perimeter-Following Function (PFF) was enabled. The variation of the inputs of the PFC (i.e., $e_{o}$ and $\dot{e_{o}}$) and the output, ω, is given in Figure 10a. According to the observed variation, the robot was able to keep the error lower most of the time. This lower error variation indicated that the robot was successful in following the perimeter. This observation can be further confirmed from the actual robot movement shown as snapshots in Figure 11a. A few occasional sudden elevations of $e_{o}$ could be observed. These elevations of $e_{o}$ were mainly due to the sudden changes of the robot heading when traversing the runners. The highest error was recorded when the robot passed a supporting structure fixed to the perimeter wall, which triggered a sudden change of the range sensors. However, the proposed PFC could cope with these sudden changes and follow the perimeter with a lower $RMS_{e_{o}}$ ($RMS_{e_{o}}$ was 2.8 cm, given in Table 3).

In Test Case b, the robot was initially placed with a heading error toward the perimeter, as shown in Figure 9b. The variation of $e_{o}$ indicated that the root had a higher positive error at the start, and it tended to zero within the next few seconds due to the PFC action of commanding a positive angular rotation (see Figure 10b). After correcting the initial error, the robot was moved almost similar to the first case, where a few sudden rises of $e_{o}$ could be observed when the robot was passing the runners. The observed movements of the robot also confirmed that the PFC was capable of successfully following the perimeter (see Figure 11b). $RMS_{e_{o}}$ was 3.5 cm, indicating a lower overall $e_{o}$. Similarly, in Case c, the robot was placed with an initial heading error outward of the perimeter. The performance and behavior of the PFC were almost similar to Case b, the only difference being the sign of the initial error. Moreover, the proposed PFC was capable of coping with initial heading errors, either inward or outward, and successfully following a perimeter.

A situation with many obstacles to be traversed by the robot was considered for Test Case d (shown in Figure 9d). Here, a few cables were placed over the surface of the false ceiling. This sort of cable lying on the surface can often be found in a false ceiling. The robot was initially placed parallel to the perimeter and commanded to follow the perimeter, similar to Case a. However, the addition of the cables created more obstacles to be traversed by the robot. According to the observed variation of $e_{0}$ (see Figure 10d), the proposed PFC was capable of maintaining a lower $e_{o}$ during the course of movement ($RMS_{e_{o}}$ = 1.4 cm). The perimeter-following ability of this case was also confirmed by the recorded movement of the robot (see Figure 11d). These results validated the ability of the PFC to correctly follow the perimeter wall while moving on rugged terrain.

In Cases e and f, the performance and behavior of the PFC in situations of inward and outward perimeter segments were evaluated (shown in Figure 9e,f, respectively). When the robot initially encountered the slanted segment, a sudden increase of $e_{o}$ could be observed for both cases. This elevated $e_{o}$ was sustained until both sensors of the robot encountered a slanted segment (around 5.5 s–8.5 s in Case e and 5.5 s–8 s in Case f). After that, the robot was capable of maintaining a lower $e_{o}$. Due to the longer sustaining of a higher $e_{o}$, the values of $RMS_{e_{o}}$ observed for the two cases were comparatively higher than the other test cases (13.0 cm and 17.2 cm for Cases e and f, respectively). However, the robot successfully followed the perimeter, as confirmed by the recorded movement of the robot given in Figure 11e,f. A similar sort of behavior could be observed in Case g, where a combination of the inward and outward slanted perimeter was presented. Here, the elevation of $e_{o}$ was lower compared to Cases e and f since the slanted perimeter segment was smoothly connected to the perimeter without a sudden change. Thus, a lower $RMS_{e_{o}}$ was observed in this case ($RMS_{e_{o}}$ = 6.6 cm). Thus, these observations confirmed the ability of the proposed PFC to effectively cope with slanted perimeters even with subsequent direction changes.

Overall, the robot with the proposed PFC was capable of effectively following the perimeter wall of a false ceiling in all the considered test cases. The test cases consisted of heterogeneous scenarios that a false ceiling inspection robot could often encounter. Furthermore, the experiments were conducted in a testbed designed per the standard specifications of a false ceiling. Therefore, it can be concluded that the proposed PFC is very adequate for a false ceiling inspection robot to establish the perimeter-following functionality essential for the inspection process.

The model-free controlling ability of the fuzzy logic was exploited in designing the proposed PFC due to the difficulty of modeling the dynamics of the required behavior. In the event of model-free controlling of the fuzzy logic, it is not possible to provide theoretical proof to guarantee stability. Thus, an analysis to guarantee stability was not conducted in this work. Nevertheless, the behavior of the proposed controller was evaluated considering a set of diverse test cases that covered most of the probable scenarios. The experimental outcomes of these test cases validated that the controller was stable and had the ability to provide the expected behavior. Therefore, it is justifiable to consider that the proposed controller has the capability to generate expected behavior even though a theoretical analysis was not provided. Furthermore, the perimeter following ability was proposed to complement the effort of a human operator in the inspection since the perimeter following solely through teleoperation causes an unnecessary overhead for the operator. A human operator should always be engaged with the inspection process since the robot is not fully autonomous. In other words, the system is a human-in-the-loop system at the present stage. The operator can use the real-time visual feedback of the robot for inspection, as well as override any control action of the robot. Therefore, the operator can take precautionary actions to ensure the robot’s safety in the case of system instability.

## 6. Conclusions

Frequent inspections are required for false ceilings to identify progressive structural defects, as well as pest infestations. The current inspection process of false ceilings heavily depends on human labor. However, the inspection of false ceilings is difficult for humans due to the unfavorable conditions such as the confined space, low lighting conditions, and possible danger. Robots have the potential to aid false ceiling inspections. However, very little work has attempted to design robots for false ceiling inspections.

A false ceiling inspection robot should be capable of moving through narrow spaces and traversing the components of a false ceiling, such as runners and lighting fixtures. This paper proposed a novel design for a false ceiling inspection robot capable of accessing confined spaces while traversing components such as runners typically found in false ceilings. Continuous teleoperation is difficult for this sort of robot since the robot heading direction often changes due to traversing objects. Therefore, the perimeter-following ability is an essential feature for a robot designed for false ceiling inspection. The robot was deployed with a Perimeter-Following Control (PFC) to realize this requirement. The PFC was developed using fuzzy logic.

A prototype of the robot design was developed, and experiments were conducted in a mock setup of a false ceiling. The first phase of the experiments confirmed the robot’s ability to effectively traverse common objects found in a typical false ceiling. The performance and behavior of the proposed PFC were evaluated in the second phase considering heterogeneous test cases faced by a false ceiling inspection robot. The experimental results confirmed that the proposed PFC effectively established the perimeter-following ability in the false ceiling inspection robot. Moreover, the results validated the usability of the proposed robot design and the PFC to improve the false ceiling inspection process. Currently, the robot is not equipped with a localization method and a fully autonomous inspection method. These are the main limitations of the robot at the present stage. The exploration of the localization within the false ceiling and the development of a vision-based autonomous inspection frame are proposed for future work. Furthermore, explorations to develop optimal path planning methods for the robot are proposed for future work.

## Figures and Tables

**Table 1:** Comparison of existing ceiling inspection robots and Falcon.

Robot	Usability Inside a False Ceiling	Locomotion Method	Autonomy	Inspection Sensors	Payload
Blimp [17]	No	Aerial vehicle	No	Monocular camera	Limited (50 g)
Ceiling walking robot [18]	No	Walking using elastomeric footpads	No	No	Yes (100 g)
Geckobot [19]	No	Walking using elastomeric footpads	No	No	Data not available
UNIclimb [20]	No	Walking using elastomeric footpads	No	No	Data not available
Wall and ceiling climbing robot [21]	No	Magnetic wheels	No	No	Yes
Falcon (proposed design)	Yes	Wheels	Perimeter following	Monocular camera	Yes (200 g)

**Table 2:** Rule base of the fuzzy logic system.

${\dot{e}}_{o}$\$e_{o}$	NH	N	Z	P	PH
NH	R	R	R	LR	M
N	R	R	LR	M	LL
Z	R	LR	M	LL	L
P	LR	M	LL	L	L
PH	M	LL	L	L	L

**Table 3:** Root mean square of $e_{o}$ ($RMS_{e_{o}}$) for the test cases.

Case	${\mathrm{RMS}}_{e_{o}}$ (cm)
(a)	2.8
(b)	3.5
(c)	5.2
(d)	1.4
(e)	13.0
(f)	17.2
(g)	6.6

## Data Availability

Not applicable.

## Supplementary Materials

The following are available online at https://www.mdpi.com/article/10.3390/s21165281/s1, Video S1.
